# High prevalence of substance use and associated factors among high school adolescents in Woreta Town, Northwest Ethiopia: multi-domain factor analysis

**DOI:** 10.1186/1471-2458-14-1186

**Published:** 2014-11-20

**Authors:** Anteneh Messele Birhanu, Telake Azale Bisetegn, Solomon Meseret Woldeyohannes

**Affiliations:** Department of Nursing, College of Medicine and Health Sciences, University of Gondar, Gondar, Ethiopia; Department of Reproductive Health, Institute of Public Health, University of Gondar, Gondar, Ethiopia; Department of Epidemiology and Biostatistics, Institute of Public Health, University of Gondar, Gondar, Ethiopia

**Keywords:** Adolescence, Alcohol, Associated factors, Cigarette, Khat, School and substance use

## Abstract

**Background:**

Substance use is a major public health concern in global settings, and is very common during adolescence period leading to physical and/or mental health complications. This study assessed the prevalence of substance use and associated factors among high school adolescents in Woreta Town, Northwest Ethiopia, 2012.

**Methods:**

A school based cross-sectional study was conducted from April 7 to April 15, 2012 amongst 684 9^th^ to 12^th^ grade high school students in the town of Woreta. Participants were selected by stratified sampling, and data were collected using an anonymous questionnaire adapted from the 2008 Community That Care Youth Survey. Bivariate and multivariate logistic regression analysis was performed to identify factors associated with substance use.

**Results:**

A total of 651 students participated in the study with a response rate of 95.2%. The current prevalence of substance use among Woreta high school students was 47.9% and life-time prevalence was 65.4%. The current and lifetime prevalence of alcohol use was 40.9% and 59% respectively. Siblings’ use of substances (AOR [95% CI]: 2.72 [1.79, 4.14]), family history of alcohol and substance use (AOR [95% CI] 2.24 [1.39-3.59]) and friends’ use of substances (AOR [95% CI] 2.14 [1.44-3.18]) were factors positively associated with substance use. On the other hand, religiosity and social skill were found to be 54% (AOR [95% CI] 0.46, [0.31-0.68]) and 39% (AOR [95% CI] 0.6 [0.40-0.91]) negatively associated with substance use.

**Conclusions:**

The prevalence of substance use amongst adolescents was high for the three substances namely alcohol, cigarette and khat with alcohol being the most common. Community norms favorable to substance use, family history of alcohol and substance use, siblings’ substance use, poor academic performance, low perceived risk of substances and friends’ use of substances had positive association with adolescent substance use while religiosity and social skills were found to have negative association with adolescent substance use. Initiate public awareness campaigns to inform adolescents and adults, particularly parents, of the risk of substance use. Developing culture friendly, gender based adolescent and family based programs and initiating public awareness are recommended to decrease substance use by adolescents.

## Background

High level of peer influence, risk taking behavior and experimentation with substances are normal developmental changes in adolescence [1]. Globally, substance use of products such as alcohol, cigarette, and khat leaves (Catha edulis) has become a major public health concern with accompanying socio-economic problems. Studies show that substance use, particularly in developing countries, has dramatically increased [2]. Substances are used and abused widely among African youth. This situation poses serious social and public health problems similar to those in most Western societies [3]. A study among Nigerian high school students indicated that lifetime prevalence of substance use was 87.3% whereas current use was 69.2% with multiple substance use being 57.4% [4]. Lifetime prevalence rate of any substance use was found to be 69.8% among college students in Kenya [5]. In Ethiopia the commonly used substances were alcohol, cigarettes, *khat* and cannabis which frequently lead to addiction [6].

Substance use is harmful leading to decreased academic performance, increased risk of contracting HIV and other sexually transmitted diseases (STDs), or psychiatric disorders such as lethargy, hopelessness, insomnia [7] and depressive symptoms [8].

Alcohol is a serious public health problem. Globally, harmful use of alcohol results in the death of 2.5 million people annually. Alcohol contributes nearly to 4% of deaths with 6.2% of all male deaths related to alcohol compared to 1.1% death of females worldwide. Annually, 320000 young people aged 15–29 years die from alcohol-related causes resulting in 9% of all deaths in that age group globally [9]. Early onset of drinking increases the likelihood of alcohol-related injuries, motor vehicle crash involvement, unprotected intercourse, and interpersonal violence [10]. Alcohol use also contributes to youth suicides, homicides and fatal injuries [11].

The World Health Organization (WHO) report shows that beer 33%, spirits 22%, others 43% and wine 2% are consumed by people whose age is 15+ in Ethiopia [12]. A study of high school students in Dire Dawa showed the prevalence of life time and current alcohol drinking is 34.2% and 19.6% respectively [13].

Khat chewing is common in Africa and mostly in countries of the horn of Africa [13–15]. Khat consumption has a negative impact on family and social life [16, 17]. It may act as a factor that exacerbates family disruption [18]. Khat is a legal drug like cigarette and alcohol in Ethiopia, openly sold at markets and chewed in streets. It has different legal status in Africa; legal in Djibouti, Kenya, Yemen and Uganda, but illegal in Tanzania and Eritrea.

Several studies show that Khat is widely used among Ethiopian adolescents. A study done among high school adolescents in Eastern Ethiopia showed that the prevalence of khat chewing was 24.2% [19]. Another study in Dire Dawa showed the prevalence of life time and current chewing of khat was 18.4% and 10.9% respectively [13].

Globally, direct tobacco smoking causes the death of more than 5 million people in a year [20] Tobacco kills more than tuberculosis, human immunodeficiency virus/Acquired Immunodeficiency syndrome (HIV/AIDS) and malaria combined. In the next two decades the annual death toll from tobacco is expected to rise to over 8 million, with more than 80% of those deaths projected to occur in low-and middle income countries [20]. Tobacco smoking is the major single known cause of non communicable diseases [21]. It is the most important risk factor for cardiovascular disease (CVD), obstructive pulmonary disease, malignancies of the respiratory and upper gastrointestinal tract, and causes death among millions of people worldwide [5].

Tobacco smoking also is becoming an important public health problem in the developing countries [21]. There is a high prevalence of cigarette smoking in Africa. The prevalence rate of cigarette use was 42.8% in Kenyan college students [5], 20.5% among 15 years old adolescents in Zambia, and even higher (37.2%) among males younger than 12 years old in Zambia [22]. Another study in Nairobi documented a 32.2% prevalence of lifetime smoking [23].

A study in Harer, eastern Ethiopia found a 12.2% prevalence of cigarette smoking among school adolescents [24]. In Addis Ababa schools 10.1% prevalence of lifetime smoking was between 10.1%-11.5%, and current prevalence was 3%-5.6% [25].

Risk and protective factors for youth substance abuse should be assessed as a prevention program [8] as they help decrease unhealthy behavior [26]. Individual protective factors, such as engagement in positive meaningful activities, positive self concept, and religious or spiritual beliefs (religiosity) [27, 28] inhibit adolescent substance use. Peer protective factors, such as positive peer role models [28] also reduce adolescent substance use. Family factors that are found to be protective for adolescent substance use are connected to family (attachment/bonding) [27–30], positive parenting style [27–30], living in a two parent family [28], higher parent education [28], and higher parental expectations about school [28, 31]. School protective factors include being connected to school (attachment/bonding) [29, 30], and caring school climate [28, 30], and community protective factors are connected to *other* positive adults (bonded/attached) [27, 28], safe, supportive, connected neighborhood [27, 28], and community rewards for prosocial involvement [32].

Risk factors associated with increased adolescents’ substance use are favorable attitudes toward the problem behavior (including low perceived-risk of harm) [33], family history of the problem behavior [27, 28], having friends who use substance [34], community laws and norms favorable to drug use, firearms, and crime [28, 33], community disorganization [32] and poverty [27, 28].

Few studies have investigated the prevalence of substance use and the factors associated with it, as well as simultaneously examined the influence of multiple domains of risk and protective factors (associated factors) for substance use among school adolescents in Ethiopia.

Identification of factors associated with substance use at multi level/domain is essential in order to guide program planning, help adolescents to adhere to protective factors and help family, peers, and the community to increase conditions for the implementation to protective factors and reducing to risk factors and for policy responses for decision makers.

The objective of this study was to assess the prevalence and associated factors of substance use among high school adolescents in Woreta Town, Northwest Ethiopia, 2012.

## Methods

This cross sectional study was conducted in Woreta Town, the capital town of Fogera Woreda, from April 7/2012 to April 15/2012. The town is located in the South Gondar Zone of the Amhara Regional State, Ethiopia. Woreta is located 625 Km Northwest of Addis Ababa, and 55 Km from the Regional capital, Bahir Dar. The Woreda population is estimated at 245,251 as of July 2011 [35]. In Woreta town there are two government primary full cycle schools called Guaya and Dudumagn as well as one secondary and higher school where the study is conducted. The school teaches students from grade 9 to 12. As of the report of the town education sector, in the academic year of 2011/2012, there were a total of 3459 students from grades 9 to 12 attending their education. The study sample was recruited from grade 9 to 12 adolescent students (14 to 19 years age group) in Woreta secondary and high school.

The dependent variable was substance use.

The independent variables were:

- **Sociodemographic Characteristics Factors** (Age, sex, grade, religion, residence, ethnicity, mother’s educational level, father’s educational level)
- **Individual – Peer Level Domain Factors** (religiosity, belief in the moral order, rebelliousness, social skills, favorable attitude toward drug use, interaction with prosocial peers, friends’ use of drugs/substances, peer rewards for antisocial behavior)
- **Family Level Domain Factors** (family or parental attachment/mother and father attachment, family opportunities for prosocial involvement, family rewards for prosocial involvement, poor family management: poor family discipline and poor family supervision, sibling substance use, family conflict, family history of alcohol and substance use, parental attitudes favorable towards drug or substance use)
- **School Level Domain Factors** (school opportunities for prosocial involvement, school rewards for prosocial involvement, poor academic performance/Academic failure, and low commitment to school)
- **Community Level Domain Factors** (community rewards for prosocial involvement, community opportunities for prosocial involvement, low neighborhood attachment, laws favorable to substance use, community norms favorable to substance use, perceived availability of substances)

### Operational definition

**Adolescence:** The period from age 14 – 19 years.

**Substance:** the three commonly used psychoactive drugs: Alcohol, cigarette and khat that produces changes in mood, thinking, feeling, and/or behavior that can cause dependence.

**Substance Use:** Taking any of the three commonly used psychoactive substances: alcohol, cigarette and/or khat in the past 30 days.

**Risk factors:** Characteristics or conditions within the individual or in the family, school or community that increase the likelihood that someone will engage in the use of alcohol, cigarette, and khat or discourage positive behavior that might prevent them [7, 26].

**Protective factors:** Factors, characteristics or conditions within the individual or in the family, school or community that increase the likelihood of positive health behaviors or outcomes or moderate and discourage behaviors that might lead to negative health outcomes [7, 26].

**Lifetime**/**Ever-use:** Adolescents’ use of the particular substance at least once in their lifetime.

**Current use/30-day use:** Adolescents’ use of the substance at least once in the 30 days prior to data collection, and is a more sensitive indicator of the level of current use of the substance.

The sample size was determined using the formula to estimate a single population proportion based on the following assumptions: level of confidence to be 95%, 4% margin of error, and the life time and 30 days prevalence of substance use for alcohol drinking was 45.7% and 26.5%, cigarette smoking 11.5% and 5.6%, and khat chewing 16% and 7.8% respectively [36]. The study was powered on the proportion for life time alcohol use. The final sample size for the study was 684 (15% non response rate).

The study was reviewed and approved by research ethics review committee of the Institute of Public Health at the University of Gondar. Permission to conduct the research among students in the school was obtained from the school principal. The purpose and the importance of the study were clearly explained and oral consent was obtained from each participant. Confidentiality of the information was maintained by using anonymous questionnaires, by keeping the data in a secured place and by adjusting conditions for the students’ privacy while filling the questionnaires. Moreover, awareness creation education was given on substance use effects and consequences to the students. There were high proportion of participants coming from the rural areas (45.6%) whose families are in most cases illiterate (37%). High school students in the study site were older than their counterparts in the developed world. In this connection, the average age of the participants was 17.25 years (SD = 1.24). This is nearly 18 years. These students are competent /capable to give informed consent. The study is also a descriptive study with no known risk to the study participants. The research ethics committee gave us the ethical clearance considering the mention issues in the local context.

Stratified random sampling technique was used to select study participants from each grade considering population proportion to sample size. Finally respondents were selected using computer generated random number.

Data were collected with a structured pre – tested self-administered questionnaire, adapted from the 2008 ‘Community That Care (CTC) Youth Survey’ for adolescent substance use and problem behaviors. Relevant modifications were made to incorporate information specific to this study and its local context, such as the use of khat. Four trained senior nursing students and two facilitators collected data.

A pre-test was conducted with 32 high school adolescents who were attending their education in 3^rd^ compound high school and Fasiledes preparatory school in Gondar and necessary modifications made prior to the main study.

The completed questionnaires were checked for clarity, consistency and completeness daily. Consequently amendments and corrections were made. In addition, 10% of the entered data were randomly selected and cross-checked for reliability and accuracy. Because of the sensitive nature of the questions asked, respondents were made to sit apart to avoid answers being seen by peers.

Data were entered and coded in EPI INFO version 3.5.3 and then exported to SPSS Version 20 for analysis. The entered data were cleaned and recoded further for construction and categorization of the variables, and descriptive and multivariate analysis. Logistic regression was used to identify associated factors with substance use. Variables with a bivariate p value less than 0.20 were fitted in to multivariate models to control for possible confounders.

## Results

### Sociodemographic characteristics

A total of 651 students were involved in the study from 684 proposed study participants with a response rate of 95.2%, of whom 55% were male. The mean age of the students was 17.25 years (SD = 1.24). The higher percentage of the respondents 354 (54.4%) were from urban backgrounds. Almost all of the adolescents were Amhara 646 (99.2%). Most students 577 (88.6%) were Orthodox Christian followed by Muslim 53 (8.1%); 241 (37%) of the mothers and 177 (27.2%) of the fathers were reported to be illiterate, with only 26 (4%) of the mothers and 62(9.5%) of the fathers with college/university degree or above (Table 1).

**Table 1:** **Socio demographic characteristics of Woreta general higher education preparatory and secondary school students by sex (N = 651) in Woreta Town, Northwest Ethiopia, May 2012**

Characteristics	Sex		
	Female, n (%)	Male, n (%)	Total, n (%)
**Age (years)**			
14-16	108(36.9)	84(23.5)	192(29.5)
17-19	185(63.1)	274(76.5)	459(70.5)
**Grade**			
9	125(42.7)	136(38)	261(40.1)
10	102(34.8)	119(33.2)	221(33.9)
11	34(11.6)	47(13.1)	81(12.4)
12	32(10.9)	56(15.6)	88(13.5)
**Religion**			
Orthodox	259(88.4)	318(88.8)	577(88.6)
Muslim	22(7.5)	31(8.7)	53(8.2)
Others	12(4.1)	9(2.5)	21(3.2)
**Residence**			
Urban	145(49.5)	209(58.4)	354(54.4)
Rural	148(50.5)	149(41.6)	297(45.6)
**Mother’s Educational level**			
Illiterate	104(35.5)	137(38.3)	241(37)
1-6 grade	99(33.8)	135(37.7)	234(35.9)
7-12 grade	41(14)	46(12.8)	87(13.4)
Technique/vocational certificate	14(4.8)	5(1.4)	19(2.9)
Some university/college diploma	25(8.5)	19(5.3)	44(6.8)
Some university/college degree	10(3.4)	16(4.5)	26(4)
**Father’s Educational level**			
Illiterate	78(26.6)	99(27.7)	177(27.2)
1-6 grade	97(33.1)	122(34.1)	219(33.6)
7-12 grade	49(16.7)	69(19.3)	118(18.1)
Technique/vocational certificate	14(4.8)	9(2.5)	23(3.5)
Some university/college diploma	24(8.2)	28(7.8)	52(8.0)
Some university/college degree	31(10.6)	31(8.7)	62(9.5)

### Prevalence of substance use

Of the total 651 school adolescents, 312 (47.9%) reported using substances currently, and 426 (65.4%) reported lifetime use of substances. The current prevalence of substance use among male adolescents was 206 (66%) and for females 106 (34%). The current prevalence of all the three substances use was 27 (4.1%) whereas the lifetime prevalence for the three substances was 107 (16.4%).

The lifetime and current prevalence of substance use among male versus female adolescents in the school was 59% & 40.9% for alcohol drinking, 22.9% & 6.8% for cigarette smoking and 34.9% and 13.8% for khat chewing. The most commonly used substance among students was alcohol followed by khat and cigarettes both for current and lifetime use.

### Factors associated with substance use

In the multivariate logistic regression analysis, substance use was associated significantly with gender, mother’s education, community norms favorable to substance use, family history of substance use, siblings’ use of substances, poor academic performance and low perceived risk of substance use, friend’s use of substances, religiosity, and social skill (Table 2).

**Table 2:** **Multivariate logistic regression of risk and protective factors for substance use among Woreta general higher education preparatory and secondary school 9th- 12th-grade students (n = 651), Woreta Town, Northwest Ethiopia, May 2012**

Risk or Protective factors		Substance Use		OR (95%CI)	
		Yes	No	Crude	Adjusted
		n(%)	n (%)		
**Socio – Demographic characteristics**					
**Age (years)**	14-16	80(25.6)	112(33)	0.70(0.50-0.98)	0.82(0.52-1.30)
	17-19	232(74.4)	227(67)	**1**	**1**
**Sex**	Female	106(34)	187(55.2)	**1**	1
	Male	206(66)	152(44.8)	2.39(1.74-3.28)	1.52 (1.03-2.25)*
**Mother’s Ednl. Level**	Illiterate	116(37.2)	125(36.9)	**1**	**1**
	1-6 grade	132(42.3)	102(30.1)	1.40(0.97-2.00)	1.36(0.88-2.12)
	7-12 grade	41(13.1)	46(13.6)	0.96(0.59-1.57)	0.80(0.44-1.47)
	Vocl. Certificate	1(0.3)	18(5.3)	0.06(0.01-0.46)	0.51(0.005-0.55)*
	Diploma	12(3.8)	32(9.4)	0.40(0.20-0.82)	0.43(0.19-0.97)*
	Degree	10(3.2)	16(4.3)	0.67(0.29-1.54)	1.05(0.38-2.85)
**Father’s Ednl. level**	Illiterate	87(27.9)	90(26.5)	**1**	**1**
	1-6 grade	113(36.2)	106(31.3)	1.10(0.74-1.63)	0.86(0.50-1.46)
	7-12 grade	64(20.2)	54(15.9)	1.22(0.77-1.96)	1.21(0.62-2.37)
	Vocl. Certificate	10(3.2)	13(3.8)	0.80(0.33-1.91)	1.05(0.29-3.81)
	Diploma	21(6.7)	31(9.1)	0.70(0.37-1.31)	1.30(0.47-3.58)
	Degree	17(5.4)	45(13.3)	0.39(0.20-0.73)	0.47(0.15-1.55)
**Community Domain factors**					
**Low neighborhood attachment**	Yes	202(64.7)	179(52.8)	1.64(1.20-2.25)	0.94(0.62-1.43)
	No	110(35.5)	160(47.2)	**1**	**1**
Community norms favorable to Substance Use	Yes	133(65.7)	107(39.2)	2.97(2.16-4.09)	1.95(1.31-2.90)**
	No	107(34.3)	206(60.8)	**1**	**1**
Perceived availability of Substances	Yes	166(53.2)	158(46.6)	1.30(0.96-1.77)	1.02(0.68-1.53)
	No	146(44.6)	181(55.4)	**1**	**1**
Community rewards for prosocial involvement	Yes	213(68.3)	285(84.1)	0.41(0.28-0.59)	0.73(0.45 1.19)
	No	99(31.5)	54(15.9)	**1**	**1**
**Family domain factors**					
Poor family management	Yes	230(73.7)	207(61.1)	1.79(1.28-2.50)	1.40(0.91-2.12)
	No	82(26.3)	132(38.9)	**1**	**1**
Parental attitude favorable to substance use	Yes	188(60.3)	118(34.8)	2.84(2.07-3.90)	1.40(0.91-2.12)
	No	124(39.7)	221(65.2)	**1**	**1**
Family history of alcohol and substance use	Yes	129(41.3)	44(13.0)	4.73(3.20-6.97)	2.24(1.39-3.59)**
	No	183(58.7)	295(87)	**1**	**1**
Sibling’s use of substances	Yes	179(57.4)	69(20.4)	5.27(3.72-7.45)	2.72(1.79-4.14)***
	No	133(42.6)	270(79.6)	**1**	**1**
Family attachment	Yes	152(48.7)	230(67.8)	0.45(0.33-0.62)	0.94(0.59-1.50)
	No	160(51.3)	109(32.2)	**1**	**1**
Family opportunities for Prosocial involvment.	Yes	155(49.7)	229(67.6)	0.47(0.35-0.65)	0.83(0.54-1.28)
	No	157(50.3)	110(32.4)	**1**	**1**
Family rewards for prosocial involvement	Yes	131(42)	212(62.5)	0.43(0.32-0.59)	1.26(0.82-1.93)
	No	181(58%)	127(37.5)	**1**	**1**
**School domain factors**					
Poor academic performance	Yes	220(70.5)	160(47.2)	2.68(1.94-3.70)	1.67(1.12-2.47)*
	No	92(29.5)	179(52.8)	**1**	**1**
Low school commitment	Yes	203(65.1)	174(51.3)	1.77(1.29-2.42)	0.92(0.59-1.42)
	No	109(34.9)	165(48.7)	**1**	**1**
School opportunities for prosocial involvement	Yes	188(60.3)	249(73.5)	055(0.39-0.76)	1.40(0.88-2.20)
	No	124(39.7)	90(26.5)	**1**	**1**
School rewards for prosocial involvement	Yes	170(54.5)	232(68.4)	0.55(0.40-0.76)	0.89(0.56-1.41)
	No	142(45.5)	107(31.6)	**1**	**1**
**Peer and individual domain factors**					
Low perceived risk of Substance Use	Yes	230(73.3)	159(46.9)	3.18(2.28-4.42)	1.73(1.15-2.60)*
	No	82(31.3)	180(68.7)	**1**	**1**
Friends use of substances	Yes	182(58.3)	85(25.1)	4.18(2.99-5.84)	2.14(1.44-3.18)***
	No	130(41.7)	254(74.9)	**1**	**1**
Peer rewards for antisocial behaviors	Yes	183(58.7)	131(38.6)	2.25(1.65-3.08)	1.18(0.79-1.76)
	No	129(41.3)	208(61.4)	**1**	**1**
Religiosity	Yes	92(29.5)	199(58.7)	0.29(0.21-0.41)	0.46(0.31-0.68)***
	No	220(70.5)	140(41.3)	**1**	**1**
Social skills	Yes	126(40.4)	231(68.1)	0.32(0.23-0.44)	0.61(0.40-0.91)*
	No	186(59.6)	108(31.9)	**1**	**1**
Belief in the moral order	Yes	212(67.9)	284(88.3)	0.41(0.28-0.60)	0.90(0.54-1.48)
	No	100(32.1)	55(16.2)	**1**	**1**
Interaction with prosocial peers	Yes	137(43.9)	207(61.1)	0.50(0.37-0.68)	0.88(0.59-1.32)
	No	175(56.1)	132(38.9)	**1**	**1**

Abbreviations: OR, Odds Ratio; CI, Confidence Interval.

Vocl. = Vocational.

Ednl. Educational.

NB.*Remained significant at P-Value <0.05.

**Remained significant at P-Value ≤0.001.

***Remained significant at P-Value <0.0001.

### Factors associated with substance use: sociodemographic

Male students were 1.52 times more likely to use substances than their female counterparts (AOR [95% CI] 1.52 [1.03, 2.25]). Students whose mothers’ had technical/vocational certificate and some university/college diploma were 49% and 57% less likely to substance use (AOR [95% CI] 0.51 [0.005, 0.55]) and (AOR [95% CI] 0.43 [0.19, 0.97]) respectively (Table 2).

### Factors associated with substance use: the four domains

Sibling’s use of substances was the strongest predictor of substance use; odds of substance use increased by almost 3 fold with sibling’s use of substances (AOR [95% CI] 2.72 [1.79-4.14]). Students with family history of alcohol and drug use had 2.24 times higher risk of using substances as compared to those students with no family history of alcohol and drug use (AOR [95% CI] 2.24 [1.39-3.59]) (Table 2).

Students who had friends that used substances had 2.14 times higher risk of using substances than those students who had no friends who used substances (AOR [95% CI] 2.14 [1.44-3.18]). Community norms favorable to substance use was 2 times more likely to lead to adolescent substance use (AOR [95% CI] 1.95 [1.31-2.90]) than community norms that were not favorable to substance use. Students who had low perceived risk of substance use were 1.73 times more likely to use substances (AOR 1.73 [95% CI] [1.15-2.60]) compared to their counterparts who had high perceived risk of substance use. Students who had poor academic performance were 1.67 times more likely to use substances (AOR [95% CI] 1.67 [1.12-2.47]) than their counter parts who had good academic performance (Table 2).

On the other hand, students who were religious were 54% less likely to use substances compared to their counterparts who were not religious. Students who had social skills were 39% less likely to use substances (AOR [95% CI] 0.61 [0.40-0.91]) compared to their counterparts who had no social skills (Table 2). The Hosmer and Lemeshow goodness-of-fit index indicated a good model fit (*X*^2^ = 12.3 & p – value = 0.137).

## Discussion

The current prevalence of substance use in this study was 47.9%, 66% in males and 34% in females. As was demonstrated in other studies [13, 18, 25, 34, 37], males had a higher prevalence of substances use than females. Higher use among males may be related to gender roles related to external/social influences and sensation seeking behavior.

The lifetime substance use prevalence in the current study was 65.4%, which was significantly higher than the prevalence found among high school students in Addis Ababa [36] and nearly similar (69.8%) to the finding among college students in Kenya [5]. Low perceived risk of substances by adolescents, community norms favorable to substance use, substances being considered as a “gateways” substance, and the change in community value system might be the possible reasons for the high prevalence.

Similarly the current prevalence of alcohol drinking was found to be 40.9%, which is higher than almost all Ethiopian studies [13, 36] and other African countries [4]. It is more than two fold higher than the result in Dire Dawa (19.6%) [13], significantly higher than the study in Addis Ababa; 26.5% [36], and more than four times higher than the study among secondary students in Nigeria 8.9% [4]. One possible explanation is the easy access and availability of alcoholic beverages in Ethiopia. Drinking alcohol is a socially acceptable as well as a behavior learned from parents and older siblings.

The lifetime prevalence of alcohol drinking in this study was 59% which was nearly two times higher than the life time prevalence in Dire Dawa (34.2%) [13]. It was also significantly higher than a study done among high school students in Addis Ababa (45.7%) [36], and more than seven times higher (9.2%) than in Nigeria among high school students in urban setting [4]. When compared to a study done in similar setting among adolescent students in Cape Town, South Africa (50.6%), the prevalence was higher in the current study [38]. Again, high prevalence may be related to drinking being socially acceptable and readily available during holidays and other festivities in the country as well in the study area. Most often the adolescents start by experimenting with the so called “gateways drugs” such as alcohol.

The current prevalence of khat chewing in this study was found to be 13.8%. This result was higher than a similar study in Dire Dawa (10.9%) [13]. The lifetime prevalence of Khat chewing in this study was 34.9% which is nearly two fold higher than the study in Dire Dawa (18.4%) [13] as well as compared to students in Saudi Arabia (21.5%) [39]. Higher khat use may be related to the fact that khat is widely cultivated and available, and also socially acceptable. Curiosity to test new things might also be a contributing factor for its use. In addition, most adolescents aspire to be popular among their peers; one way to achieve this is through the use of substances.

The current prevalence of cigarette smoking in this study was 6.8% which was slightly higher than the study in Dire Dawa (5.6%) [13] but more than 2 times higher than among adolescents in Addis Ababa (2.9%) [34] and among high school students (3%) [11]. The life time prevalence of smoking in this study was 22.9% which is lower when compared to high school students in Nairobi (32.2%) [23]. Students in our study reported higher lifetime prevalence than the study in Dire Dawa, Ethiopia (13%) [13], and more than 2 times higher than the study in Addis Ababa 10.1% [11]. Again, this study was slightly higher than the 16.9% rate reported among teenage students in a city of South Brazil [40]. Easy access and availability, and curiosity to try new things may be reasons for the higher rates in our study. The absence of legal provision to control the spread cigarette aggravates the use in the area.

Substance use was associated significantly with gender of the students. The current study was similar to results in Addis Ababa [34]. These indicate that males are at greater risk of substance use, perhaps due to social norms and gender roles.

Students whose mothers’ had technical/vocational certificate and some university/college diploma have a lower risk of substance use, respectively, consistent with findings among African American students on tobacco use [41].

Siblings’ use of substances emerged as the stronger predictor of substance use, with a nearly 3 fold increase among students who reported having siblings’ who used substances. Findings from other studies on adolescent substance use in South Africa have also documented an association between substance use by other members of the household and adolescent substance use [38], indicated use may be a learned behavior.

The current study revealed that there is association between low perceived risks of substance use and substance use. Students with low perceived risks of substance use were 1.73 times more likely to use substances compared to their counter parts with high perceived risk of substance use. Studies in Maine and Oregon in the U.S. also found similar results. Low perceived risk of substance use is more strongly associated with the use of alcohol and cigarettes [42]. Adolescents who don’t know the dangers of substance use may be more likely to use them.

Family history of alcohol and substance use was risk factors for substance use. Studies with high school students in Ethiopia also revealed an association between family history of alcohol and substance use and substance use [34].

The study in Dire Dawa showed fathers/guardians use of alcohol and mother/female guardians’ use of alcohol were significantly associated with substance use [13].

A study in South Africa among high school adolescents also documented similar evidence; substance use by other members of the household is significantly associated with substance use [38]. Similarly, in Zambia, parental smoking had positive association with adolescents’ substance use [22]. The finding is also consistent with the cross-national comparisons of risk and protective factors for adolescents; substance use in United States, Australia, Brazil and the U.S. [40, 42–44].

These all studies give additional evidence that family history of alcohol and substance use leads to a learned behavior among children regarding substance use.

Friends’ use of substances was also found to be predictor of substance use, with students who had friends that used substances had 2.14 times higher risk of using substances than those students who had no friends that used substances. Similar results were reported for smoking in Addis Ababa [37]. This finding is further evidence of the impact of social norms and learned behaviors on adolescents’ use of substances. Peer pressure is very powerful factor for influencing behavior especially in young people. Adolescents who affiliate with substance use peers may be pressured to use substances.

Students in our study with poor academic performance were at higher risk of using substances when compared with students with good academic performance, consistent with findings among adolescents in the U.S. and Australia for cigarette and alcohol use [42, 44], among Ethiopian high school students [36].

Community norms favorable to substance use was found to be a predictor for substance use, with nearly 2 times the odds of using substances in areas with community norms favorable to substance use, similar to findings in Australia [42, 44]. Reasons could be related to exposure to such behaviors in various media (films and TV), commercial values of substances, and permissive law of the country on substance use.

On the other hand, protective factors such as religiosity and social skills were associated with lower substance use. Being religious decreased the risk of substance use by 54%, consistent with past studies [27, 28, 44]. Religious teachings discourage the use of substances and may, thus, protect people from engaging in self destructive behaviors including substance use.

Having social skills reduced the risk of substance use by 39%, consistent with findings in the past studies for alcohol and drug use (43, 46). Adolescents with effective communication skills may better withstand peer pressure to use substances.

Although some research suggests that community opportunity for prosocial involvement is a protective factor for adolescent substance use [42], the current study did not show a significant association. One possible justification for the discrepancy might be differences in measurement and the difference in the socio-demographic status of the samples across studies.

In addition, poor family management was a strong predictor of adolescent substance use in other research [42, 44] but in our study. However, this finding should not be taken to suggest that poor family management is not related to substance use in adolescents. Instead, research must be done to clarify the association of family and community factors among adolescents using larger samples and across wider geographical areas, and in longitudinal/cohort studies.

### Limitations & strengths of the study

Social desirability bias may have affected participants’ response. Prevalence rates were also self-reported and dependent on the accuracy with which the participants recalled and reported such use. Additionally, the cross-sectional design only allows for description of behavior at one point in time. A major strength of our study is that results are largely consistent with past literature, and increasing generalizability of our findings.

## Conclusions

The prevalence of substance use among high school adolescent students in Woreta was high for all three substances: alcohol, cigarette and khat. Alcohol was the most common substance used among adolescents. The prevalence of substance use was found higher among male adolescent students.

Norms favorable to Substance use (community domain), family history of alcohol and drug use and sibling’s substance use (family domain), poor academic performance (school domain), low perceived risk of substances and friend’s use of substances (individual – peer domain) were risk factors which were found to have positive association with substance use. Being a male was also associated with substance use.

Both religiosity and social skills from individual-peer domain were protective factors which were found to have inverse or negative association with adolescent substance use. Having technique/vocational certificate and some University/College diploma of mothers had also negative association with adolescent substance use.

### Recommendations

Overall, several factors contributed to increased substance use, and could be focused on in education to decrease risky behaviors among adolescents.

Multi-dimensional prevention programming fostering anti-substance use attitudes among individual adolescents, parents and peers may be most effective in preventing and reducing substance use patterns among school adolescents by the government. Content should be culturally appropriate and gender targeted, and family focused. Interventions to decrease use among adolescents could be focused on leveraging protective factors such as religiosity and social skills, and decreasing the negative influence of norms favorable to substance use in the community domain and parents, siblings and friends’ substance use, improving academic performance, and increasing perceived risk of substances use. Given the number of influences on adolescent substance use, such a multi-factorial approach may be most impactful. Public awareness campaigns to inform adolescents and adults, particularly parents, of the risk of substance use maybe initiated through diverse media formats. Education in primary and secondary schools could be integrated into curricula and strengthened with special focus on the adverse consequences of the substances.

## Authors’ information

1. AM is Lecturer, Department of Nursing, College of Medicine and Health Sciences at University of Gondar, Ethiopia. AM has Bachelor of Science Degree in Nursing and Masters Degree in Public Health-Reproductive Health.
2. TA is Assistant Professor of Public Health, Department of Reproductive Health, Institute of Public Health at University of Gondar, Ethiopia. TA has Diploma in Nursing, Advanced Diploma in Psychiatry Specialize Nursing, Bachelor of Science Degree in Public Health Officer, Masters Degree in Public Health and A PHD candidate in Psychiatry Epidemiology.
3. SM is an Assistant Professor of Biostatistics, Department of Epidemiology and Biostatistics, Institute of Public Health, University of Gondar, Ethiopia. SM has Bachelor of Science Degree in Statistics, Masters Degree in Public Health - Biostatistics and Epidemiology and A PHD candidate in Public Health.

## Abbreviations

AIDS: Acquired immunodeficiency syndrome
CSA: Central Statistical Agency of Ethiopia
CVD: Cardiovascular diseases
DACA: Drug Administration and Control Authority of Ethiopia
HIV: Human immunodeficiency virus
STD: Sexually transmitted diseases
US: United States
WHO: World Health Organization.
